# Nocturnal melatonin increases glucose uptake via insulin-independent action in the goldfish brain

**DOI:** 10.3389/fendo.2023.1173113

**Published:** 2023-05-23

**Authors:** Kazuki Watanabe, Masaki Nakano, Yusuke Maruyama, Jun Hirayama, Nobuo Suzuki, Atsuhiko Hattori

**Affiliations:** ^1^ Department of Biology, College of Liberal Arts and Sciences, Tokyo Medical and Dental University, Ichikawa, Chiba, Japan; ^2^ Department of Clinical Engineering, Faculty of Health Sciences, Komatsu University, Komatsu, Ishikawa, Japan; ^3^ Department of Sport and Wellness, College of Sport and Wellness, Rikkyo University, Niiza, Saitama, Japan; ^4^ Division of Health Sciences, Graduate School of Sustainable Systems Science, Komatsu University, Komatsu, Ishikawa, Japan; ^5^ Noto Marine Laboratory, Institute of Nature and Environmental Technology, Kanazawa University, Noto-Cho, Ishikawa, Japan

**Keywords:** melatonin, N1-acetyl-5-methoxykynuramine (AMK), glucose homeostasis, glucose uptake, brain, diurnal rhythm

## Abstract

Melatonin, a neurohormone nocturnally produced by the pineal gland, is known to regulate the circadian rhythm. It has been recently reported that variants of melatonin receptors are associated with an increased risk of hyperglycemia and type 2 diabetes, suggesting that melatonin may be involved in the regulation of glucose homeostasis. Insulin is a key hormone that regulates circulating glucose levels and cellular metabolism after food intake in many tissues, including the brain. Although cells actively uptake glucose even during sleep and without food, little is known regarding the physiological effects of nocturnal melatonin on glucose homeostasis. Therefore, we presume the involvement of melatonin in the diurnal rhythm of glucose metabolism, independent of insulin action after food intake. In the present study, goldfish (*Carassius auratus*) was used as an animal model, since this species has no insulin-dependent glucose transporter type 4 (GLUT4). We found that in fasted individuals, plasma melatonin levels were significantly higher and insulin levels were significantly lower during the night. Furthermore, glucose uptake in the brain, liver, and muscle tissues also significantly increased at night. After intraperitoneal administration of melatonin, glucose uptake by the brain and liver showed significantly greater increases than in the control group. The administration of melatonin also significantly decreased plasma glucose levels in hyperglycemic goldfish, but failed to alter insulin mRNA expression in Brockmann body and plasma insulin levels. Using an insulin-free medium, we demonstrated that melatonin treatment increased glucose uptake in a dose-dependent manner in primary cell cultures of goldfish brain and liver cells. Moreover, the addition of a melatonin receptor antagonist decreased glucose uptake in hepatocytes, but not in brain cells. Next, treatment with N1-acetyl-5-methoxykynuramine (AMK), a melatonin metabolite in the brain, directly increased glucose uptake in cultured brain cells. Taken together, these findings suggest that melatonin is a possible circadian regulator of glucose homeostasis, whereas insulin acquires its effect on glucose metabolism following food intake.

## Introduction

Blood glucose and insulin levels dramatically change after food intake, but basal insulin levels—as well as basal glucose concentrations— vary throughout the day (1–3). In humans, basal insulin levels at night are reportedly lower than those during the day (4, 5). Energy is required for cell survival even during sleep when the level of plasma insulin is lower. Notably, glucose uptake by the human brain during sleep is comparable to or even higher than when awake (6, 7). The factors responsible for this phenomenon have been hypothesized as acting independently of insulin, which is primarily regulated by food intake.

Melatonin (N-acetyl-5-methoxytryptamine) is known as the “hormone of darkness” since it is synthesized by the pineal grand at night based on the length of the period of darkness (8), and because it is involved with the circadian clock (9). Although primarily synthesized by the vertebrate pineal gland, melatonin is also synthesized by other tissues including the skin (10) and retina (11). It is also an ancestral hormone present in unicellular organisms (12) and cyanobacteria (12) as well as in plants (13), invertebrates (12), and vertebrates including fish (14, 15). In addition, melatonin metabolites including 3-hydroxymelatonin (3-OHM), 2-hydroxymelatonin (2-OHM), N1-acetyl-N2-formyl-5-methoxykynuramine (AFMK), or N1-acetyl-5-methoxykynuramine (AMK) have been detected in mammals (16, 17), invertebrates (18), and plants (19). Melatonin receptors belong to the G protein-coupled receptor family and are found in invertebrates (20), vertebrates and plants (20). Humans and other mammals have two melatonin receptor subtypes, MTNR1A (MT1) and MTNR1B (MT2). In mammals, melatonin receptors are present in the brain, suprachiasmatic nucleus, retina, spleen, spinal cord, intestine, kidney, prostate, ovary, skin, fat, muscle, and liver, implying the widespread physiological actions of melatonin via these receptors. Moreover, melatonin can inhibit the binding of calcium to calmodulin (21), and its functions as an endogenous free-radical scavenger and broad-spectrum antioxidant are well established (22). Taken together, these findings demonstrate that melatonin has diverse physiological functions, which are thought to be supported by evolutionary relationships.

Recent research findings have suggested the involvement of melatonin function in glucose metabolism in concert with insulin in mammals. The physiological action of insulin, which decreases plasma glucose levels and increases glucose uptake in adipocytes, myocytes, brain cells, and hepatocytes, is well understood in mammals. Glucose transporter type 4 (GLUT4) is an insulin-regulated glucose transporter that is primarily found in adipose tissue, skeletal muscle, brain, and liver in mammals. Despite extensive phylogenetic analysis and comprehensive database searches, no orthologs of mammalian *glut4*, which is responsive to insulin, have been found in lancelets, lampreys, hagfish, zebrafish, or goldfish. Moreover, to date, no reports including the terms of “goldfish” and “glut4” were found using the NIH PubMed search engine. These findings suggest that functional *glut4* arose as a glucose transporter during late vertebrate evolution (23, 24). Therefore, the importance of insulin in energy metabolism has been questioned for the teleost (25, 26).

In the present study, we aimed to elucidate the physiological role of melatonin in diurnal variation of glucose metabolism. For this purpose, we used goldfish, which are genetically close to zebrafish with no identified Glut4 gene and are suitable for *in vivo* experiments. In addition, research using goldfish has been previously undertaken to investigate the relationship between the pineal grand and glucose metabolism by many researchers, beginning with Delahunty et al. (27–29). First, for *in vivo* experiments, we used individuals placed in a fasted state to examine diurnal changes in glucose uptake in the brain as well as liver and muscle, and whether melatonin administration altered these values. Next, in an *in vitro* experiment, goldfish brain and liver cells were cultured under insulin-free culture conditions to examine the direct effects of melatonin and its brain metabolite, AMK, on glucose incorporation.

## Materials and methods

### Animals

Adult goldfish (*Carassius auratus*, about 10–15 cm) were purchased from a commercial source (Higashikawa Fish Farm, Nara, Japan) and maintained under normal laboratory conditions (i.e., 24 ± 1°C, 12hrs light: 12hrs darkness, lights on 8:00 AM–8:00 PM). Prior to the start of the *in vivo* experiment, goldfish were subjected to a 24h period of fasting. For day and night experiments, one-third of goldfish (n = 6 per group) were subjected to light exposure (3500 lux) during the night for three days before the experiments. All experimental procedures were conducted in accordance with the Tokyo Medical and Dental University Guidelines for the Care and Use of Laboratory Animals.

### Plasma collection

To collect plasma, goldfish were first anesthetized with MS-222 (Sigma-Aldrich, St Louis, MO, USA), and 100 µl of whole blood was collected from the vertebral vein using a 1 ml heparinized syringe with a 21-gauge needle (Terumo Corporation, Tokyo, Japan) at mid-day (2:00 PM, 3500 lux) or mid-night (2:00 AM, dark phase, 20 lux under dim red light or light exposure condition, 3500 lux) (n = 6). Plasma samples were prepared by centrifugation at 1000 × g for 10 min at 4°C and were then stored at −80°C for further analysis.

### 2DG uptake in the brain, liver, and muscle tissues

Incorporated 2-deoxyglucose (2DG; Sigma-Aldrich), a glucose molecule in which the 2-hydroxyl group is replaced by hydrogen, is converted to 2DG-6-phosphate (2DG6P) by intracellular metabolic processes. Because 2DG6P accumulates in cells due to the absence of downstream metabolizing systems, glucose uptake can be estimated by measuring 2DG6P level. After 24 h of fasting, goldfish were intraperitoneally injected with 2DG (330 µg/g bw) dissolved in 0.9% saline at mid-day (i.e., 2:00 PM) or mid-night (2:00 AM, dark phase or light exposure condition) (n = 6). Brain, liver, and muscle tissues were collected 30 min after 2DG injection and were then homogenized in ultrapure water. In primary experiments, we confirmed that incorporated 2DG reached its maximum levels 60 min after 2DG administration (**Figure S1**). Samples were then centrifuged at 20,000 × g for 10 min at 4°C and the supernatant was stored at −80°C until 2DG6P measurement.

### Measurement of insulin, glucose, melatonin, and 2DG6P

Plasma insulin levels were determined by an enzyme-linked immunosorbent assay kit (ELISA) with a fish-specific antibody. All procedures were performed according to the manufacturer’s instructions (Fish Insulin ELISA Kit, Cusabio Biotech Co., Wuhan, China). Insulin content was determined using 50 μl plasma for each assay. Plasma glucose levels were measured by using the LabAssay Glucose Kit (Wako Pure Chemical Industries) based on the mutarotase GOD method; all procedures were performed as per the manufacturer’s instructions. Plasma glucose levels were determined using 2 μl plasma for each assay.

To measure melatonin and 2DG6P levels, we employed an LC-MS-8050 system, which is a triple quadrupole liquid chromatograph mass spectrometer (LC-MS/MS; Shimadzu, Kyoto, Japan) coupled with an LC-30AD high performance liquid chromatography system (Shimadzu) and a SIL-30AC autosampler (Shimadzu). For 2DG6P measurement, we used an amide-80 column (i.e., 2.0 mm × 150 mm, 3.0 µm particle size; Tosoh Bioscience LLC) with a column oven temperature of 25°C. The isocratic elution consisted of 10 mM ammonium acetate with 0.05% acetic acid and methanol (3:1, v/v). The flow rate was set to 0.4 ml/min. To detect the melatonin content, we used an ODS (OctaDecylSilyl) column (i.e., 2.0mm × 150 mm, 3.0 µm particle size; Tosoh Bioscience LLC, King of Prussia, PA, USA) with a column oven temperature of 25°C. The flow rate of the mobile phase was 0.3 ml/min. Once again, a gradient was used with a mobile phase of 10 mM ammonium acetate with 0.05% acetic acid and 100% methanol. The gradient began with 5% methanol and increased to 50% methanol within 20 min.

Quantification was performed using a mass spectrometer equipped with an electro spray ionization source in the multiple reaction monitoring mode. Melatonin and 2DG6P were monitored in the positive ionization mode using transitions of m/z 233.00 → m/z 130.00 and m/z 243.15 → m/z 96.75, respectively. The collision energies were −43 eV and −25 eV, the Q1 Pre Bias values were −25 V and −15 V, and the Q2 Pre Bias values were −23 V and −16 V, respectively. To confirm the identity of the compounds, melatonin and 2DG6P were monitored by other transitions of m/z 233.00 → m/z 159.10 (for melatonin) and m/z 243.15 → m/z 78.90 (for 2DG6P). LC solution software (Shimadzu) was used for instrument control and data acquisition.

### *In vivo* 2DG uptake in the brain, liver, and muscle tissues, and plasma insulin levels following melatonin injection

Goldfish were subjected to a 24 h fasting period prior to 2DG injection. 2DG (330 µg/g bw) was dissolved in 0.9% saline and injected intraperitoneally along with melatonin (8 ng/g bw) or vehicle (n = 6). Brain, liver, and muscle tissues were removed 30 min after injection and homogenized in ultrapure water. Samples were then centrifuged at 20,000 × g for 10 min at 4°C and supernatant was collected. At the same time, whole blood was collected to obtain plasma. The supernatant and plasma samples were stored at −80°C for further measurement of 2DG6P and insulin content, respectively.

### Measurement of plasma glucose and insulin levels following melatonin injection

To investigate the effect of melatonin on glucose homeostasis under induced hyperglycemic conditions, glucose (330 µg/g bw) and melatonin (8 ng/g bw) were simultaneously administered into the peritoneal cavity. Blood samples were then collected 0, 20, 60, and 120 min after administration during the daytime. Plasma glucose levels were measured in the control, glucose-, and glucose+melatonin treatment groups. The dose-dependent effect of melatonin on plasma glucose levels was examined following the intraperitoneal injection of glucose (330 µg/g bw) and melatonin (0.08, 0.8, and 8 ng/g bw), and both assessments were performed in the same way (n = 6–7). Plasma samples were collected from the three treatment groups (n = 5 per group) 20 and 60min following administration and were then stored at −80°C until insulin measurement. The Brockmann body (i.e., the principal sites of insulin production in fish (30)) were collected 120 min after injection and total RNA was isolated (n = 6–7).

### RNA isolation and complementary DNA synthesis

First, samples of Brockmann body, brain, liver, and muscle tissues were collected from goldfish during the daytime. Total RNA was then isolated from these tissues using an ISOGEN kit (Nippon Gene, Tokyo, Japan); all procedures were performed as per the manufacturer’s instructions. Next, 1 µg of total RNA was reverse-transcribed to cDNA using PrimeScript Reverse Transcriptase and oligo dT primers (Takara Bio, Shiga, Japan).

### Measurement of changes in insulin mRNA expression in the Brockmann body

To assess changes in the mRNA expression levels of insulin in the goldfish Brockmann body quantitative real-time PCR (qPCR) was performed using primers for insulin and β-actin as an internal standards (**Table 1**). qPCR amplification was then carried out using an Mx3000P qPCR System (Agilent Technologies, Santa Clara, CA, USA) and SYBR Ex *Taq* DNA Polymerase II (Takara Bio). The amplification conditions comprised 40 cycles of denaturation at 95°C for 10 s and annealing/extension at 65°C for 40 s followed by melt curve analysis. The mRNA expression level of insulin was normalized to that of β-actin.

**Table 1:** Primers used in the present study.

Real-time PCR primers	Sequence
Insulin-F	5’-CGTCTCCGGTGTAAATGCTAACG-3’
Insulin-R	5’-TGGAGGAAGGAAACCCAGAAGAG-3’
β-actin-F	5’-CGAGCGTGGCTACAGCTTCA-3’
β-actin-R	5’-GCCCGTCAGGGAGCTCATAG-3’
Real-time PCR primers	Sequence
Mel1a 1.4-F	5’-CCATCGGCATCGTCACGTACT-3’
Mel1a 1.4-R	5’-CACCGCCAGGCCTATGAAGTT-3’
Mel1a 1.7-F	5’-TGGGCTTTGACGGTGCTTG-3’
Mel1a 1.7-R	5’-ACCAGAACCCAGATTCGCAAGT-3’
Mel1b-F	5’-CTCCTGCACCTTCACGCAGA-3’
Mel1b-R	5’-CGCAGGTCGCTGGGTCTTAC-3’
Mel1c-F	5’-GGTGGTGGCGCTGTACCC-3’
Mel1c-R	5’-GCGTCCAGGTGAGCAGCA-3’
GLUT1-F	5’-TCTTCCGCTCATCGCTCTACC-3’
GLUT1-R	5’-GGCTCAGACACTCCTGCTTTCTC-3’
GLUT2-F	5’-CATCGCATTAGCTGGGTGTTG-3’
GLUT2-R	5’-AAAGGGTGAAACCGAACAGGAG-3’
β-actin-F	5’-CGAGCTGCGTGTTGCCCCTGAG-3’
β-actin-R	5’-CGGCCGTGGTGGTGAAGCTGTAG-3’

### 2DG incorporation in cultured primary brain cells and hepatocytes

Cell cultures of goldfish brain cells were created as previously described (31, 32). Briefly, cells from whole brains (viability ≥90%) were prepared by trypsin digestion then cultured in 24-well poly-L-lysine coated plates at 6.0 × 10^5^ cells/ml/well with Neurobasal medium (Gibco) supplemented with 200 mM L-glutamine and 2% B27 supplement (Gibco), which contains no insulin. Goldfish hepatocytes (viability ≥95%) were prepared using the collagenase digestion method with minor modifications (33) before being cultured in serum-free DMEM/F-12 (Gibco), which also contains no insulin, in 24-well plates at 1.0 × 10^6^ cells/ml/well. Cells were maintained in 28°C (5% CO_2_ with saturated humidity) for three days for brain cells and 24 h for hepatocytes prior to use in downstream experiments. Brain cells underwent a starvation treatment with glucose-free Neurobasal medium (Gibco) for 6h prior to 2DG uptake experiment. We confirmed this starvation condition was not affected cell morphology and few adherent cells were floated. The medium was then replaced with 2DG medium, which contained 2DG (1 mM) in glucose-free Neurobasal medium, with or without added melatonin (i.e., 0.1 nM, 10 nM, and 1 µM; Wako Pure Chemical Industries, Osaka, Japan). Next, cells were collected at either 30 or 60 min after the 2DG and melatonin treatment (n = 6–10). For primary experiments, we confirmed that the incorporated 2DG6P was detectable in brain cells when using a medium that contained at least 1 mM 2DG, and the value of 2DG6P was increased up to this time point.

For the melatonin receptor antagonist experiment in hepatocytes, the medium was replaced with 2DG medium that also contained 10 nM melatonin and 1 µM Luzindole (Sigma-Aldrich) and was then cultured for 60 min (n = 6). Cultured hepatocytes were also subjected to 6 h of starvation in a glucose-free DMEM/F-12 mixed medium. This medium was then replaced with a 2DG medium that contained 2DG (10 mM) in glucose-free DMEM/F-12 mixed medium and was supplemented with different concentrations of melatonin (i.e., 1 nM, 100 nM, and 10 µM). Next, cells were collected at two time points—i.e., 30, or 60 min—following the 2DG and melatonin treatment (n = 6–10). As before, in primary experiments, we confirmed that incorporated 2DG6P was detectable in hepatocytes by using a medium containing at least 10 mM 2DG. For another melatonin receptor antagonist experiment in brain cells, the medium was replaced with 2DG medium that contained 100 nM melatonin and 10 µM Luzindole before the cells were cultured for 60 min (n = 6). As before, cultured brain cells were first starved, then the starvation medium was replaced with a medium 2DG (1 mM) and AMK (0.1 nM) followed by further culturing for 30 or 60 min. The salvaged cells from this experiment were lysed using a microtip-sonicator (Sonics Vibra Cell, Basingstoke, UK) and centrifuged at 20,000 × g for 10 min at 4°C. The supernatant obtained was then stored at −80°C until 2DG6P measurement.

### mRNA expression of melatonin receptors and glucose transporters in the brain, liver, and muscle tissues

Four subtypes of melatonin receptors (i.e., Mel_1a_1.4, Mel_1a_1.7, Mel_1b_, and Mel_1c_) are expressed in goldfish (34). We examined the mRNA expression patterns of these melatonin receptors as well as glucose transporters (i.e., Glut1 and Glut2) in the goldfish brain, liver, and muscle tissues. Analyses were performed using reverse transcription polymerase chain reaction (RT-PCR). Samples were collected in the daytime. PCR amplification was conducted using Ex *Taq* DNA Polymerase (Takara Bio) and primers specific to each mRNA of interest (see **Table 1**). The amplification conditions were as follows: denaturation at 95°C for 50 s followed by 40 cycles of 95°C for 10 s, 65°C for 30 s, and 68°C for 1 min. Next, PCR products were separated by electrophoresis on 2.5% agarose gels that were then stained with ethidium bromide before being visualized under UV light illumination. β-actin was used as an internal amplification control; no amplified products were found when using the reverse-transcription-omitted samples as a negative control.

### Statistical analyses

Data are presented as mean ± standard error. The means of more than two groups were compared using analysis of variance (ANOVA). Multiple comparisons were evaluated using Tukey’s honest significant difference tests (Tukey’s HSD) or Dunnett’s multiple comparison tests. Differences between the treatment and control groups were assessed using Student’s *t*-tests. A *P* values < 0.05 was considered statistically significant.

## Results

### Plasma melatonin, insulin, and glucose levels at mid-day, mid-night, and influence of nocturnal light exposure

**Figure 1:**
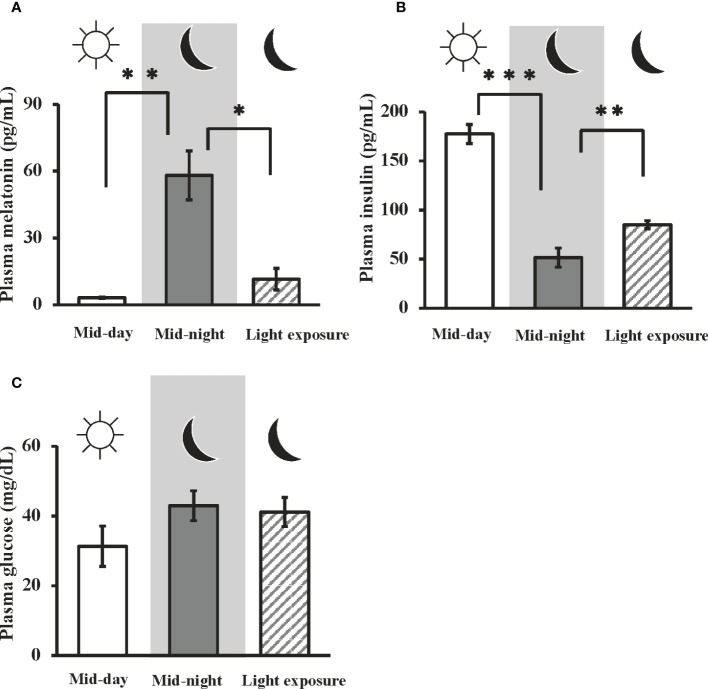
Day-night changes in plasma melatonin, insulin, and glucose levels, and the influence of nocturnal light exposure. In goldfish, plasma concentrations of **(A)** melatonin, **(B)** insulin, and **(C)** glucose were measured by a LC-MS/MS system, ELISA, and commercial kit, respectively. Samples were collected at mid-day, mid-night, and mid-night under the nocturnal light (3500 lux) exposure condition (n = 6). *P < 0.05, **P < 0.01, and ***P < 0.001 indicate significant differences vs. mid-night levels.

In goldfish, the plasma melatonin levels at mid-night were significantly higher than that at mid-day (i.e., 3.17 ± 0.272 pg/ml at mid-day, and 58.1 ± 11.0 pg/ml at mid-night). Moreover, nocturnal light exposure was found to attenuate nocturnal melatonin secretion (11.6 ± 4.80 pg/ml) (**Figure 1A**). On the other hand, plasma insulin levels were significantly decreased from mid-day to mid-night (i.e., 177 ± 9.63 pg/ml at mid-day, 51.6 ± 9.77 pg/ml at mid-night), but this effect was significantly restored by nocturnal light exposure (85.1 ± 4.10 pg/ml) (**Figure 1B**). No significant change in plasma glucose levels were observed among the three groups (**Figure 1C**).

### *In vivo* 2DG uptake in the goldfish brain, liver, and muscle tissues at mid-day, mid-night, and influence of nocturnal light exposure

The mRNA expression profiles of melatonin receptors and glucose transporters mRNA in the goldfish brain, liver, and muscle tissues are shown in **Figure S2**. Most mRNAs melatonin receptors were expressed in all three tissues. In contrast, Glut1 mRNA expression was detected in the brain and muscle, whereas Glut2 mRNA was observed only in brain and liver tissues.

**Figure 2:**
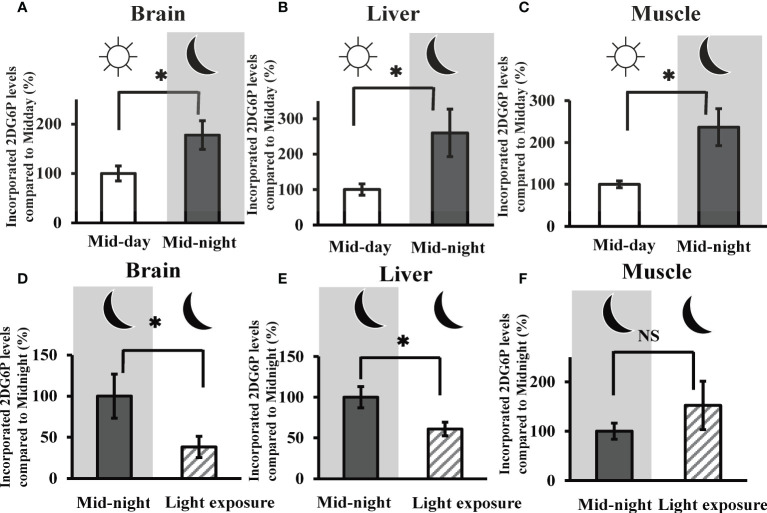
*In vivo* 2DG uptake in the goldfish brain, liver, and muscle tissues at mid-day, mid-night, and the influence of nocturnal light exposure. The incorporated amounts of 2DG6P in the **(A)** brain, **(B)** liver, and **(C)** muscle tissues were determined 30 min after intraperitoneal injection of 2DG (330 µg/g bw) at mid-day and mid-night using the LC-MS/MS system (n = 6). The influence of nocturnal light exposure (3500 lux) on 2DG6P incorporation into the **(D)** brain, **(E)** liver, and **(F)** muscle tissues was assessed in the same manner (n = 6). *P < 0.05 indicates significant differences.

Given the time course of 2DG uptake (i.e., measuring 2DG6P levels) in the brain, liver, and muscle tissues, our results showed a liner increase in uptake values for the first 60 minutes (**Figure S1**). Then, in subsequent experiments, we evaluated 2DG uptake in the goldfish brain, liver, and muscle tissues at 30 min after 2DG administration. In all three tissues, 2DG uptake was significantly augmented at the mid-night time point (i.e., 5850 ± 951 pmol/mg brain tissue, 11.5 ± 2.93 pmol/mg liver tissue, and 110.7 ± 20.7 pmol/mg muscle tissue) compared to mid-day (> 177% vs. mid-day) (**Figures 2A–C**). Nocturnal light exposure was found to significantly decrease 2DG uptake by the goldfish brain (**Figure 2D**) and liver (**Figure 2E**) tissue (i.e., < 61.0% vs. mid-night), but this decrease was not observed in muscle tissue (**Figure 2F**).

### *In vivo* effect of melatonin on 2DG uptake in goldfish brain and liver tissue, and plasma insulin levels

**Figure 3:**
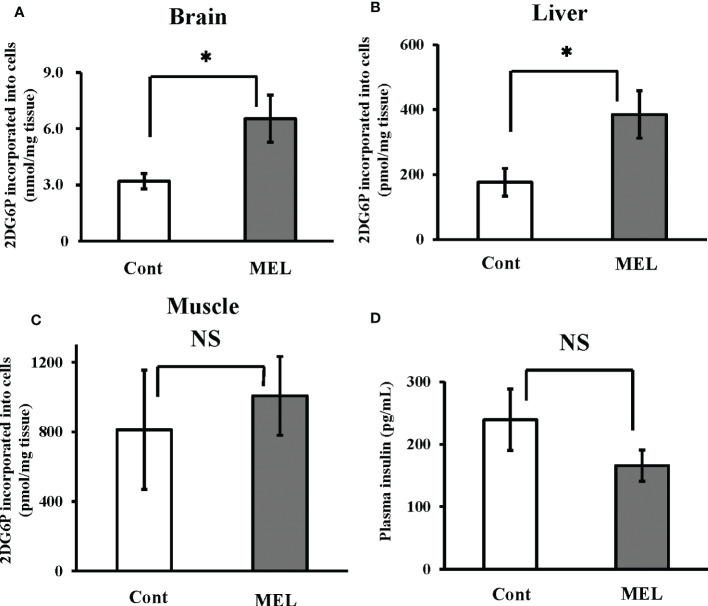
*In vivo* effects of melatonin injection on 2DG uptake in the goldfish brain, liver and muscle tissues, and plasma insulin levels. 2DG uptake into the **(A)** brain, **(B)** liver, and **(C)** muscle tissues was determined 30 min after intraperitoneal co-administration of 2DG (330 µg/g bw) and melatonin (8 ng/g bw; MEL) or vehicle (Cont) during the daytime (n = 6). **(D)** At the same time, plasma samples were collected and insulin levels were measured. *P < 0.05 indicates significant differences.

Thirty minutes after intraperitoneal co-administration of 2DG (330 µg/g bw) and melatonin (8 ng/g bw), 2DG uptake in the brain and liver showed a significantly greater increase than uptake (6.53 ± 1.26 nmol/mg brain tissue, 385.3 ± 73.1 pmol/mg liver tissue) in the group administered only 2DG (i.e., 204% and 218% vs. the 2DG-only group, respectively) (**Figures 3A, B**). In contrast, melatonin failed to alter 2DG uptake in the muscle tissue (**Figure 3C**). Plasma insulin levels 30 min after melatonin administration were also unchanged compared to the control group (**Figure 3D**).

### Melatonin Injection and plasma glucose and insulin levels, and insulin mRNA expression under induced hyperglycemic conditions

**Figure 4:**
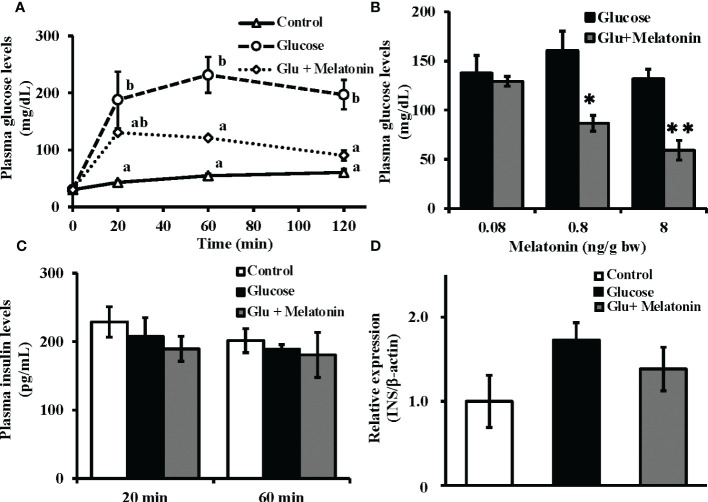
Suppressive effect of melatonin under induced hyperglycemic conditions on plasma glucose levels without alteration of plasma insulin and insulin mRNA levels in the Brockmann body. **(A)** Time courses of plasma glucose levels in goldfish under induced hyperglycemic conditions. Glucose (330 µg/g bw) and melatonin (8 ng/g bw) were intraperitoneally administered during the daytime. Blood samples were collected before and 20, 60, and 120 min after administration and plasma glucose levels were measured (n = 5). Groups with different letters showed significant differences (*P* < 0.05). **(B)** The dose-dependent effect of melatonin on plasma glucose levels was assessed 120 min after injection of glucose and melatonin (n = 6–7). *P < 0.05 and **P < 0.01 indicate significant differences vs. the glucose-only treatment group. **(C)** Plasma insulin levels at 20 and 60 min (n = 5) and **(D)** insulin mRNA expression in the Brockmann body at 120 min (n = 6–7) after administration of glucose (330 µg/g bw) and melatonin (8 ng/g bw) were also determined. No significant changes were observed in plasma insulin levels or insulin mRNA expression.

After intraperitoneal co-administration of glucose (330 µg/g bw) and melatonin (8 ng/g bw), significant decreases in plasma glucose levels were observed relative to the group that had received only glucose (**Figure 4A**). Furthermore, melatonin administration (0.08, 0.8, and 8 ng/g bw) lowered plasma glucose levels 120 min after injection in a dose-dependent manner (**Figure 4B**). However, both the glucose-only administration and its co-administration with melatonin failed to alter plasma insulin levels after 20 and 60 min (**Figure 4C**). Moreover, no significant changes in insulin mRNA expression levels in the Brockmann body were found 120 min after glucose-only injection or after co-administration with melatonin (**Figure 4D**). Relative to initial mRNA levels, significant changes in insulin mRNA expression were observed at 4, 6, and 12 h after glucose-only administration (**Figure S3**).

### Effects of melatonin and Luzindole on 2DG incorporation by cultured primary brain cells and hepatocytes, and the effect of AMK on 2DG incorporation using an insulin-free medium

**Figure 5:**
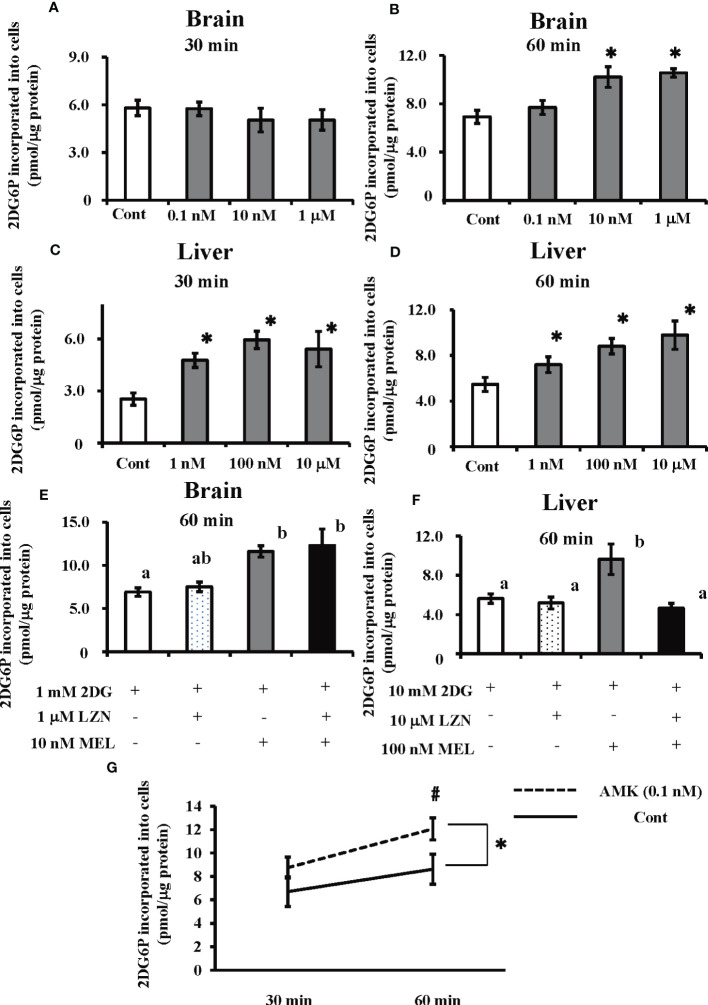
Effects of melatonin and Luzindole treatment on 2DG incorporation by cultured primary goldfish brain and liver cells, and the effect of AMK on 2DG incorporation by cultured primary brain cells using an insulin-free medium. After co-treatment with 2DG and melatonin, 2DG6P levels in the cultured primary brain cells were determined **(A)** 30 min and **(B)** 60 min later and in hepatocytes **(C)** 30 min and **(D)** 60 min later (n = 6–10). *P < 0.05 indicates significant differences vs. the 2DG-only treatment group (Cont). The effects of melatonin (MEL) and Luzindole (LZN) on 2DG incorporation into **(E)** brain cells and **(F)** hepatocytes were assessed 60 min after treatment (n = 6–10). Groups with different letters showed significant differences (*P* < 0.05). **(G)** After co-treatment with 2DG and melatonin metabolite AMK, 2DG6P levels in the brain cells were determined at 30 and 60 min later. *P < 0.05 indicates significant differences between control group (Cont) and AMK treated group compared by ANOVA. ^#^ *P* < 0.05 indicates significant differences in 2DG incorporation between the two groups after 60 min.

The amount of 2DG6P incorporated into cultured brain cells was found to significantly increase following melatonin treatment at 60 min (i.e., 10 nM and 1 µM), whereas 30 min of treatment did not have a significant effect (**Figures 5A, B**). In cultured hepatocytes, both 30 and 60 min melatonin treatments (i.e., 1 nM, 100 nM, and 10 µM) significantly augmented 2DG incorporation into cells (**Figures 5C, D**). Luzindole, a melatonin membrane receptor antagonist, did not alter the action of melatonin on 2DG incorporation into cultured brain cells; however, in cultured hepatocytes, increased 2DG incorporation was abolished by co-treatment of melatonin with Luzindole (**Figures 5E, F**). Treatment with AMK (0.1 nM), a melatonin brain metabolite, in cultured brain cells significantly increased the incorporation of 2DG relative to the control group (p < 0.05). Moreover, there was a significant (p < 0.05) difference in 2DG incorporation between the two groups after 60 min (**Figure 5G**).

## Discussion

Interestingly, glucose intolerance, insulin resistance, and other alterations associated with metabolic disorders in humans can be seen in physiological and pathological states as related to reduced melatonin levels. These can be due to aging, shift work, and exposure to light during the night (35–37). In addition, associations of MT2 variants with hyperglycemia and/or increased risk of type 2 diabetes have been reported (38, 39). Thus, impaired melatonin signaling is postulated to play a role in diabetes risk, and melatonin treatment ameliorates glucose metabolism in pathological states such as diabetes and obesity.

As shown in **Figure 1**, melatonin levels are elevated at night and insulin levels rise during the day. Therefore, it is likely that both hormones regulate the basal level of blood glucose concentration. Intraperitoneal administration of melatonin was found to increase brain and liver 2DG uptake (**Figures 3A, B**), while nocturnal light exposure results in reduced 2DG uptake in the brain and liver (**Figures 2D, E**). Moreover, this treatment also reduced plasma melatonin levels and increased plasma insulin levels (**Figures 1A, B**). Our results suggest that glucose uptake in the brain and liver was upregulated by melatonin increases during the night, while in other tissues was upregulated by insulin secretion at day or by light exposure condition independently of food intake. To maintain vital activity, circulation glucose concentrations were managed strictly by many factors. Although we found that plasma glucose levels were unaffected by light conditions (**Figure 1C**), other hormones may be involved in the control of glucose levels. Several hormones are involved in glucose homeostasis including thyroxine, and the addition of artificial lights at night did not alter plasma thyroxine in the Eurasian perch (40). Although melatonin and insulin are altered by light exposure, it is conceivable that thyroxine and other hormones unaffected by light exposure may be somehow involved in glucose homeostasis.

Interestingly, plasma glucose concentrations show a circadian rhythm in diurnal rodents as well as in nocturnal mice and rats, However, this glucose rhythm is oppositely phased between nocturnal and diurnal animals (41). Plasma glucose in diurnal animals shows a daily rhythm, with a peak before the beginning of the wake period and the pattern for nocturnal animals is similar diurnal animals and shows a peak before the dark period. If samples were collected at only mid-day and mid-night, there may be no significant differences among mammals. On the other hand, the uptake of 2DG in muscle tissue was found to be unaffected during melatonin depletion by light exposure (**Figure 2F**) or by intraperitoneal melatonin administration (**Figure 3C**). In general, mammalian muscle is a main target of insulin action, so insulin-sensitive glucose transporters (mainly GLUT4). Reduction in the Glut4 content of plasma membrane in adipose tissue was observed in pinealectomized rats (42). Several reports have demonstrated that melatonin treatment improves insulin resistance in skeletal muscle (43, 44). Therefore, melatonin may be involved in glucose uptake in some tissues, including muscle, which related to Glut4 in mammals. The relationship between melatonin and insulin in the muscle is expected to be a complex mechanism, but further investigation of this relationship between them will clarify the role of melatonin in glucose metabolism.

Despite the expression of all melatonin membrane receptor mRNAs examined by this study in goldfish brains (**Figure S2**), the increase in 2DG incorporation following melatonin treatment was not suppressed by the melatonin antagonist Luzindole (**Figure 5E**). A positive correlation between melatonin and AMK levels was also observed in the brains of goldfish following melatonin treatment (**Figure S4A**). Moreover, we also found that a low dose (0.1 nM) of the melatonin metabolite AMK can directly increase 2DG incorporation in cultured brain cells (**Figure 5G**). Thus, the effect of melatonin on brain glucose uptake does not appear to be mediated by melatonin membrane receptors. However, to date we do not have any data regarding cell type because we could not obtain the suitable antibodies for neural markers of goldfish. However, using a poly-l-lysine-coated substrate, TB2 cells (isolated from adult tilapia brain tissue) showed increased in neuronal dopamine decarboxylase (DDC) and microtubule-associated protein 2 (MAP2) (45). Therefore, neuron may account for large percentages of all brain cells present. Furthermore, our results showed that *in vivo* injection of melatonin increased 2DG uptake in the brain after 30 min (**Figure 3A**). This indicated that, as in mice (46), the plasma concentrations of this melatonin metabolites following melatonin injection increased within 5 min compared to before melatonin administration (**Figure S4B**). Thus, we speculate that AMK, a metabolite of melatonin, plays an important role in brain glucose homeostasis via a melatonin receptor-independent pathway. In contrast, melatonin injection enhanced 2DG uptake in the liver (**Figure 3B**), and melatonin treatment enhanced 2DG incorporation in cultured goldfish hepatocytes in a dose-dependent manner (**Figures 5C, D**). Importantly, we also found that this effect was prevented by co-treatment with Luzindole (**Figure 5F**). Luzindole is supposed to be an antagonist of melatonin; however, some reports have showed that it can exert paradoxical effects (47). It may also act as a partial agonist of the melatonin receptor subtype found in human kidney cells (48) and have similar effects in rats (49, 50). Luzindole has also been found to act as a partial antagonist or a full agonist depending on the receptor subtype in medaka (51). In a previous *in vivo* experiment in goldfish, Luzindole administration before melatonin blocked the melatonin-induced events (52). Our results consistent with the hypothesis that Luzindole blocks glucose uptake in hepatocytes caused by melatonin treatment. Here, the expression profile of melatonin receptor mRNA, coupled with other results, suggest that melatonin regulates to regulate glucose uptake via membrane receptors in the liver.

The action of melatonin is mediated via melatonin receptors, which belong to the G protein-coupled receptor superfamily. Three melatonin receptors MTNR1a (MT1/Mel_1a_), MTNR1b (MT2/Mel1_b_), and MTNR1c (Mel_1c_), have been identified in vertebrates. MTNR1a and MTNR1b have been identified in many different vertebrate species investigated, whereas Mel_1c_ is typically only found in non-mammalian species. In addition, MTNR1a (MTNR1a1/MTNR1a1.7) and MTNR1a-like (MTNR1a2/MTNR1a1.4) have been identified as two distinct MTNR1a subtypes that are found in teleostean species (34, 51, 53, 54). Fish-specific whole-genome duplication events are thought to have occurred in the teleostean lineage (55). The four paralogs MTNR1a, MTNR1b, MTNR1c and MTNR1d (MTNR1a-like) were thought to have been generated after the second genome duplication event. Our results showed that Mel1a1.7 (MTNR1a) was only expressed weakly in the liver. Because melatonin was found to have increased glucose uptake in cultured liver cells and that this phenomenon was blocked by the melatonin antagonist, the effect of melatonin may be exerted via MTNR1b, MTNR1c and MTNR1d.

We found that intraperitoneal injection of melatonin significantly lowered the blood glucose levels relative to administration of glucose alone (**Figure 4A**). However, this treatment did not change the insulin levels in the blood (**Figure 4C**) or the mRNA levels of insulin in the Brockmann body, which is the main insulin-producing organ in fish(**Figure 4D**). In a previous study, insulin mRNA levels in the Brockmann body were unchanged 2 h after glucose administration (**Figure S3**). This result supports previous reports carried out on teleosts (25, 26). Two hours after glucose administration, if insulin levels do not change, melatonin significantly reduces the blood glucose level. Melatonin was also found to have increased glucose uptake in the brain and liver during the night, when endogenous insulin levels are low (**Figure 1E**). This result indicates that melatonin may act as a hypoglycemic hormone like insulin. It is thus possible that melatonin—rather than insulin—may play a crucial role in glucose homeostasis in lower vertebrates. Future studies are needed to investigate the effects of melatonin on nocturnal glucose metabolism in many mammals, including humans.

The sugar transporter family is found in bacteria, archaea, and eukaryotes. Their metabolic function is to provide uniport or proton-coupled symport modes of transport (56). The cyanobacterium *Synechocystis* sp. PCC 6803 can utilize glucose as an externally supplied carbon source for growth. *Synechocystis* uptakes external glucose via a GlcP (glucose transporter) and then further metabolizes it via glycolysis and the oxidative pentose phosphate pathway (57, 58). The glucose transporter found in *Staphylococcus epidermidis* (GlcP_Se_), which is specific to glucose, shows high sequence homology to human GLUT and is inhibited by human GLUT inhibitors (58). Moreover, facilitative glucose transporter 1 (FGT-1) is the major—and maybe the only—GLUT-like protein present in *Caenorhabditis elegans* (*C. elegans)*. Several reports have noted that FGT-1 is closely related to the mammalian GLUT2-like intestinal glucose transporter (59, 60). Thus, glucose can be thought of as the primary energy source of life on earth, with the glucose transport system being an essential part of living cells. On the other hand, melatonin and/or melatonin metabolites are also present in cyanobacterium (12), plants (13, 19), and invertebrates including *C. elegans* (61). Moreover, melatonin receptors exist in many invertebrates(e.g., crayfish (62), honeybees (63), *C. elegans* (61), among many others) and plants (19). Thus, it would be interesting to study whether glucose regulation is an ancestral function of melatonin and its metabolites in these taxa.

To the best of our knowledge, this study of goldfish is the first to demonstrate that nocturnal melatonin directly regulates glucose uptake in place of insulin in a vertebrate species. Further studies are required to investigate the direct effects of melatonin on glucose regulation (i.e., separate from diet) in vertebrates.

## Data availability statement

The original contributions presented in the study are included in the article/**Supplementary Material**. Further inquiries can be directed to the corresponding author.

## Ethics statement

The animal study was reviewed and approved by Animal Care Committee of the Experimental Animal Center at Tokyo Medical and Dental University (A2022-038A).

## Author contributions

KW and MN performed most of the experiments. YM provided advice on measurement of 2DG uptake using the LC-MS/MS system. JH assisted in some studies and reviewed the manuscript. NS provided the cDNA database of goldfish. AH supervised all studies and drafting of the manuscript.
