# Supplementary material for: Nocturnal melatonin increases glucose uptake via insulin-independent action in the goldfish brain
Source: Front Endocrinol (Lausanne). 2023 May 23;14:1173113. doi: 10.3389/fendo.2023.1173113 (PMC10242130; doi:10.3389/fendo.2023.1173113)
Supplement: Supplementary file 1 [file DataSheet_1.pdf]

Supplementary Material

Nocturnal melatonin increases glucose uptake via insulin-independent action in goldfish brain

Kazuki Watanabe, Masaki Nakano, Yusuke Maruyama, Jun Hirayama, Nobuo Suzuki, and Atsuhiko Hattori*.

*** Correspondence: Atsuhiko Hattori**: ahattori.las@tmd.ac.jp

Supplementary Figures

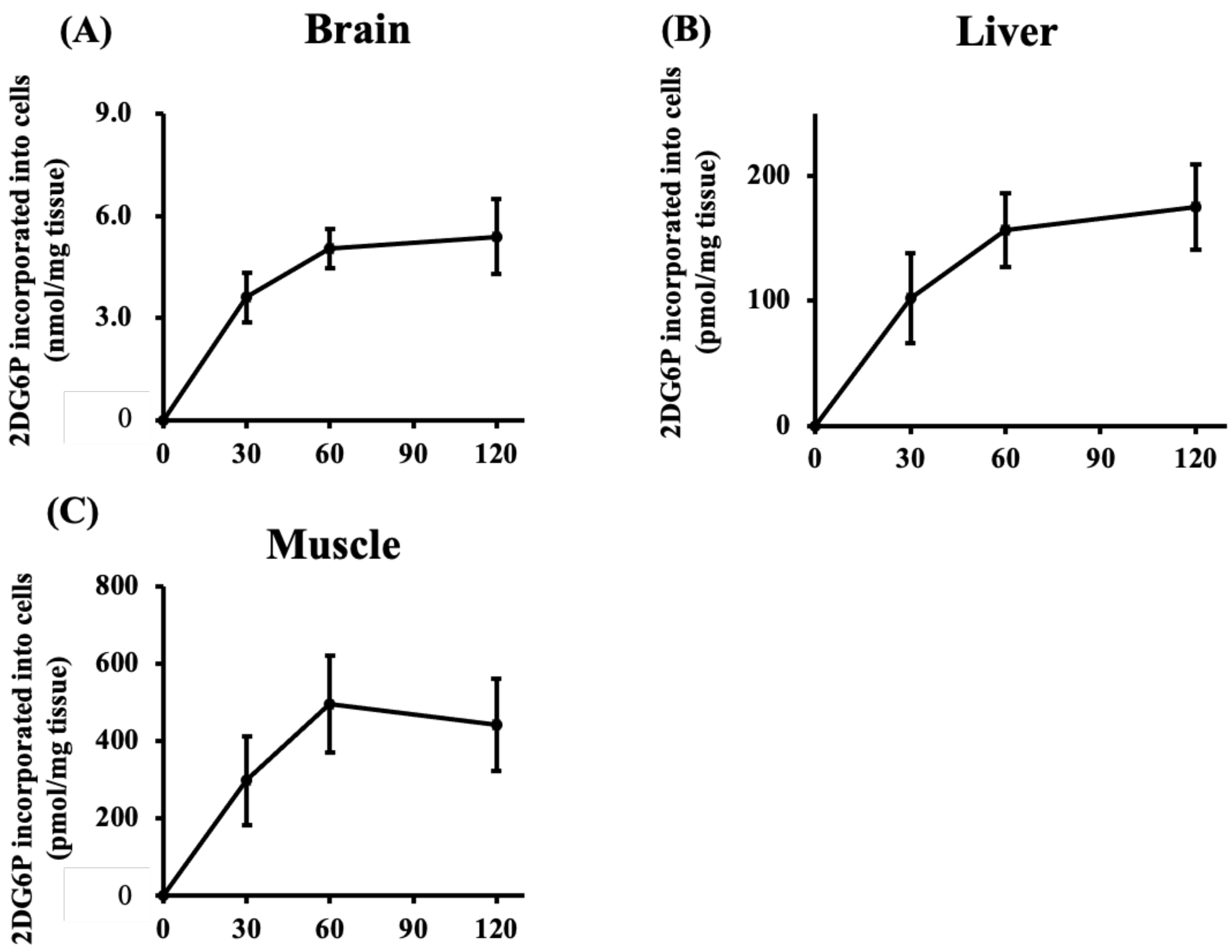

Supplementary Figure S1. Time course of 2DG uptake in the goldfish brain, liver and muscle tissues.

2DG6P levels in (A) brain, (B) liver, and (C) muscle tissues were determined at 30, 60 and 120 min after administration of 2DG (330 µg/g bw) (n = 6–8).

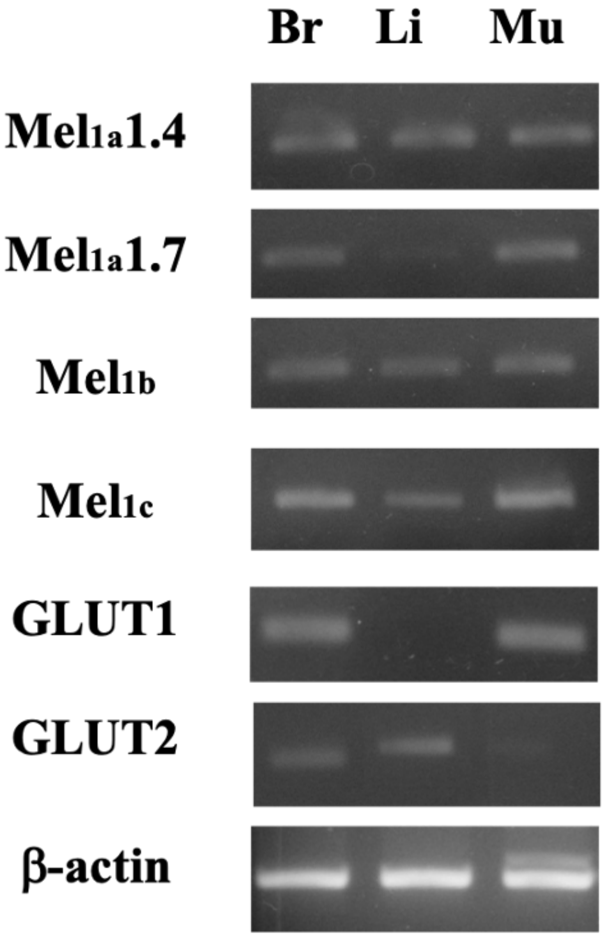

Supplementary Figure S2. Expression pattern of melatonin receptors and glucose transporters mRNA in goldfish tissues.

Expression profiles of melatonin receptors (Mel_{1a}1.4, Mel_{1a}1.7, Mel_{1b}, and Mel_{1c}) and glucose transporters (Glut1 and Glut2) mRNA in goldfish brain (Br), liver (Li), and muscle (Mu) tissues were analyzed by RT-PCR and agarose gel electrophoresis.

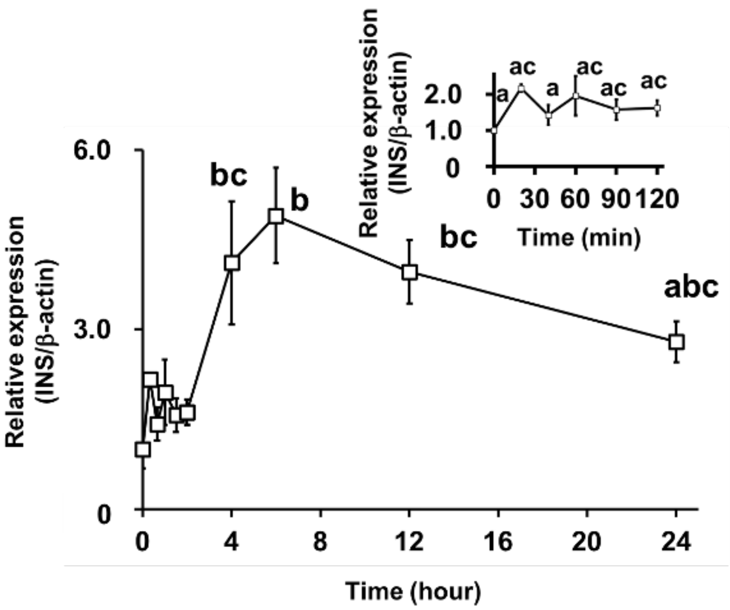

Supplementary Figure S3. Time course of insulin mRNA expression levels in the goldfish Brockmann body after glucose loading.

Insulin mRNA expression in the Brockmann body 0, 20, 40, 60, 90, 120 min, 4, 6, 12, and 24 h after glucose administration (330 µg/g bw) was determined by qPCR (n = 4). The mRNA expression level of insulin was normalized to that of β-actin. The insert (upper right) shows an enlarged image up to

120 min after glucose administration. Groups with different letters showed significant differences ($P < 0.05$).

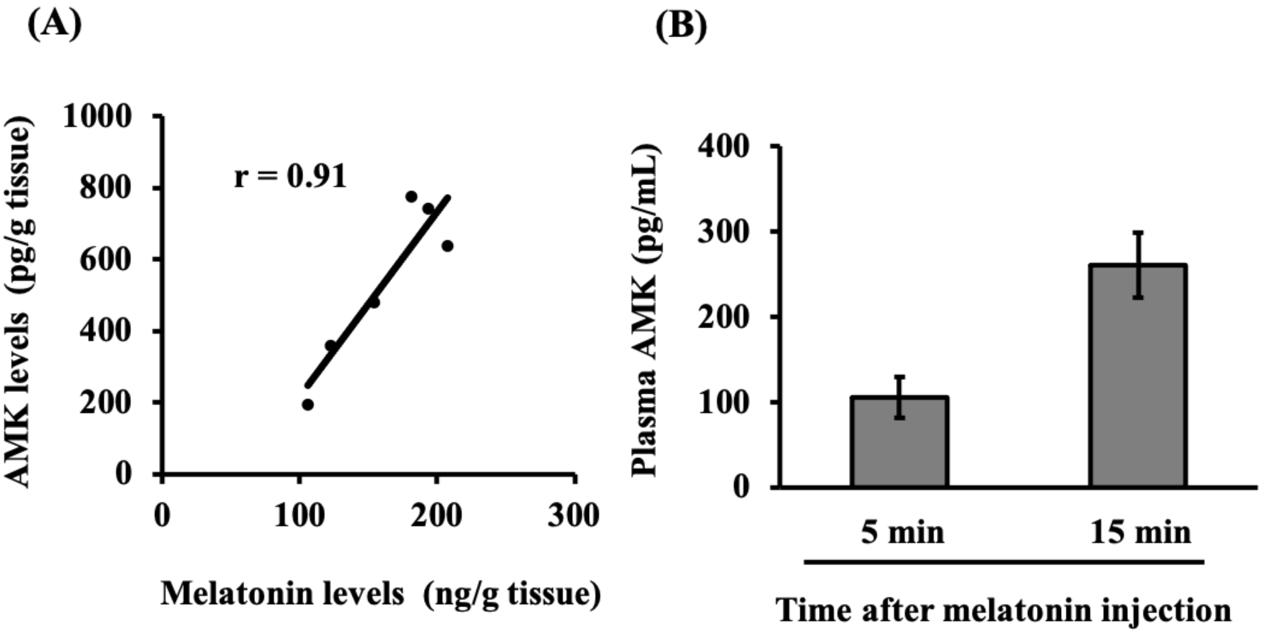

Supplementary Figure S4. Melatonin and AMK relationship in goldfish brain after melatonin injection.

(A) Correlations between melatonin and AMK levels of the brain of goldfish at 15–60 min after melatonin injection. (B) Plasma AMK levels in goldfish after melatonin (800 ng/g bw) injection. Plasma AMK levels before injection were below detection limits.
